# Respiratory muscle training in stroke patients with respiratory muscle weakness, dysphagia, and dysarthria – a prospective randomized trial: Erratum

**DOI:** 10.1097/MD.0000000000020194

**Published:** 2020-04-24

In the article, “Respiratory muscle training in stroke patients with respiratory muscle weakness, dysphagia, and dysarthria – a prospective randomized trial”^1^, which appears in Volume 99, Issue 10 of *Medicine*, in the second paragraph of the introduction “>90%” should be “<90%.” In that same paragraph, force expiratory flow should be forced expiratory flow.

Table 7 appeared twice as Tables 7 and 8. The correct Table 8 appears below.

**Table 8:**
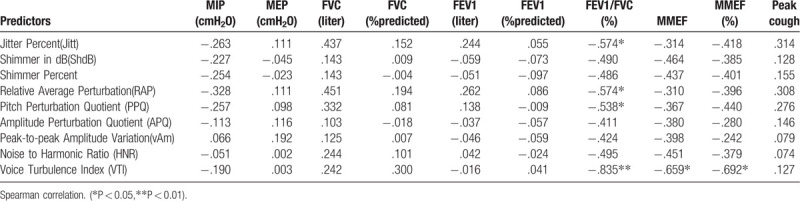
Relationships between Multi–Dimensional Voice report and cardiopulmonary function.

